# A global view of shifting cultivation: Recent, current, and future extent

**DOI:** 10.1371/journal.pone.0184479

**Published:** 2017-09-08

**Authors:** Andreas Heinimann, Ole Mertz, Steve Frolking, Andreas Egelund Christensen, Kaspar Hurni, Fernando Sedano, Louise Parsons Chini, Ritvik Sahajpal, Matthew Hansen, George Hurtt

**Affiliations:** 1 Institute of Geography, University of Bern, Bern, Switzerland; 2 Centre for Development and Environment, University of Bern, Bern, Switzerland; 3 Department of Geosciences and Natural Resource Management, University of Copenhagen, Copenhagen K, Denmark; 4 Institute for the Study of Earth, Oceans, and Space, University of New Hampshire, Durham, United States of America; 5 Department of Geographical Sciences, University of Maryland, College Park, United States of America; Montana State University Bozeman, UNITED STATES

## Abstract

Mosaic landscapes under shifting cultivation, with their dynamic mix of managed and natural land covers, often fall through the cracks in remote sensing–based land cover and land use classifications, as these are unable to adequately capture such landscapes’ dynamic nature and complex spectral and spatial signatures. But information about such landscapes is urgently needed to improve the outcomes of global earth system modelling and large-scale carbon and greenhouse gas accounting. This study combines existing global Landsat-based deforestation data covering the years 2000 to 2014 with very high-resolution satellite imagery to visually detect the specific spatio-temporal pattern of shifting cultivation at a one-degree cell resolution worldwide. The accuracy levels of our classification were high with an overall accuracy above 87%. We estimate the current global extent of shifting cultivation and compare it to other current global mapping endeavors as well as results of literature searches. Based on an expert survey, we make a first attempt at estimating past trends as well as possible future trends in the global distribution of shifting cultivation until the end of the 21^st^ century. With 62% of the investigated one-degree cells in the humid and sub-humid tropics currently showing signs of shifting cultivation—the majority in the Americas (41%) and Africa (37%)—this form of cultivation remains widespread, and it would be wrong to speak of its general global demise in the last decades. We estimate that shifting cultivation landscapes currently cover roughly 280 million hectares worldwide, including both cultivated fields and fallows. While only an approximation, this estimate is clearly smaller than the areas mentioned in the literature which range up to 1,000 million hectares. Based on our expert survey and historical trends we estimate a possible strong decrease in shifting cultivation over the next decades, raising issues of livelihood security and resilience among people currently depending on shifting cultivation.

## 1. Introduction

Recent international efforts to compare and synthesize different earth system models have come with a strong focus on quantifying the past, current, and future contributions of land use to climate change [1–4]. However, adequate prediction of land use–based emissions requires an improved understanding of megatrends in land use systems change [2,5]. While the literature offers good representations of the major natural land covers and human land uses [6–8], mosaic landscapes with a dynamic mix of managed and natural land covers often fall through the cracks in global land cover and land use classifications, as these are unable to adequately capture such landscapes’ dynamic nature and complex spectral signatures [9–12]. This has led to a paucity of global information on certain land use systems, including shifting cultivation at the global level. To date, we know little about its worldwide extent, underlying spatial patterns, or global trends in its past and future development. This became particularly evident when Hurtt et al. [2] included shifting cultivation in a global harmonization of land use states and transitions from past to future: they found only one (hand-drawn) global map of shifting cultivation, in a book on economic geography dating from 1980 [13]. At the same time, shifting cultivation was one of the most sensitive variables in their model runs (along with wood harvesting). Accordingly, they emphasized that “the need for global data on annual global gridded land-use transitions from past-to-future presents a large and underdetermined problem” [2].

Besides the need to determine the effects of shifting cultivation on land use–based greenhouse gas emission scenarios, there are other important reasons for gaining a better understanding of change in shifting cultivation systems. Shifting cultivation has often been blamed as the main cause of deforestation and forest degradation [9–11,14,15], but evidence is growing that when shifting cultivation is discontinued, it is often replaced by intensified land uses with higher environmental impacts [16,17]. For example, many of the commercial or smallholder oil palm and rubber plantations that cover large areas of Southeast Asia today are on land that was formerly used for shifting cultivation [18–21].

Lastly, it is also problematic that shifting cultivation has been subject to recycling of statements about its importance that have no basis in thorough empirical research. Nobody knows how many people today depend on shifting cultivation globally [22]. A review focusing on Southeast Asia found little aggregate information about the areas under shifting cultivation there [10], and we were unable to find information about shifting cultivation areas in Africa and Latin America.

Our main objectives in this study are therefore 1) to review published knowledge about current status and past trends in the development of the global extent of shifting cultivation; 2) to assess the recent global distribution of shifting cultivation and, based on these trends and expert statements, 3) to provide a first estimation of the future extent and spatial distribution of shifting cultivation until 2090. This will be useful in improving the characterization of land surface and land use dynamics for earth system models and large-scale carbon and greenhouse gas accounting. Our point of departure is a global map of the distribution of “primitive subsistence agriculture” produced by Butler in 1980 [13], a visual inspection of the distribution of shifting cultivation based on the 2000–2014 Global Forest Change (GFC) data set [8] and very high–resolution satellite imagery, as well as an expert survey. Our predictions of future extents of shifting cultivation are, of course, speculative. In fact, they should be understood as “best guesses” about general patterns rather than temporally and spatially accurate predictions, as land use transitions often happen suddenly, causing abrupt changes over large areas [23]. But developing predictions is essential to estimating future land use–based greenhouse gas emissions, and we consider that our approach will help to improve existing projections, which essentially assume the area under shifting cultivation to remain constant in the future [2]. The main outcomes presented in this study are maps showing the estimated presence of shifting cultivation at a one-degree resolution for the present, as well as, for the first time, estimations for 2030, 2060, and 2090. The maps focus on the tropical parts of Central and South America, Africa, South and Southeast Asia, and the Southwest Pacific for two reasons: 1) These areas have the most biomass, causing land use transitions in these areas to have a particularly high impact on global carbon emissions; and 2) shifting cultivation is most widespread in these areas today [17].

## 2. Methods

### 2.1 Available literature on the current global extent of shifting cultivation

To assess published scientific material on the current extent of shifting cultivation, we searched the Web of Science (“All Databases”) using the following search string:

[Title]: "shifting cultivation" or swidden* or "slash and burn" or "slash-and-burn" or "shifting agriculture" AND [Year published]: 2005–2016.

The search was performed in January 2016 and generated 324 articles, which we then screened for data on numbers or estimates of global or national areas influenced by shifting cultivation. Articles with data at subnational scales were only considered if the subnational area studied constituted the main area of shifting cultivation in the given country (and thus a reasonable estimate of the national extent of shifting cultivation). We limited the search to the period from 2005 to 2016 partly because we were interested in the most recent data on the extent of shifting cultivation as a basis for generating a map showing the contemporary situation (around the year 2010); the other reason was that we expected many recent reports on areas under shifting cultivation to rely on previously published data, which would enable targeted backtracking through the literature all the way to the original sources. In addition to searching the Web of Science, we also consulted three major book publications that could be assumed to contain relevant information [24–26].

### 2.2 Visual assessment of current landscapes with signs of shifting cultivation

**Fig 1:**
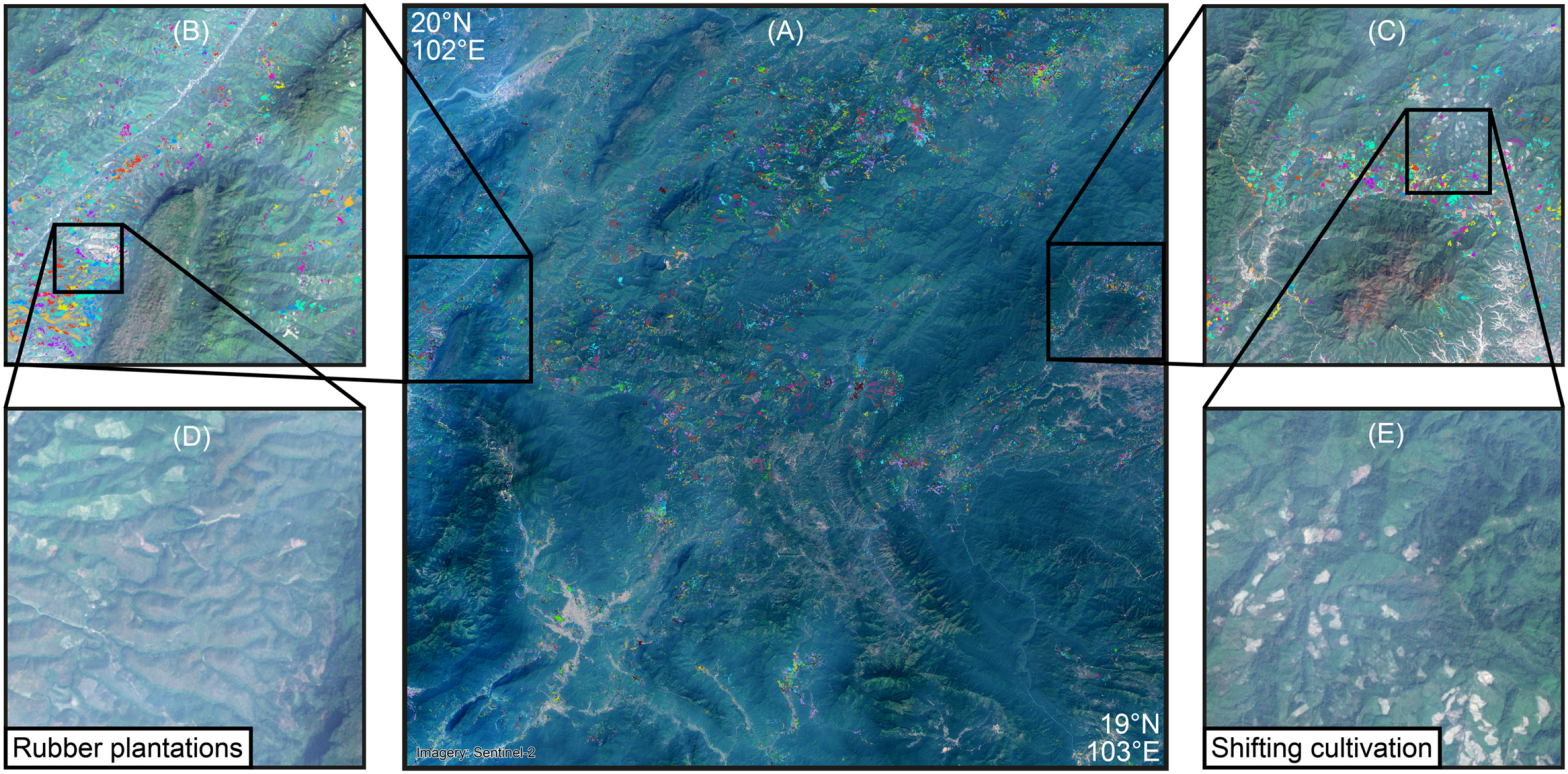
Identification of spatio-temporal pattern based on GFC global annual deforestation data [8] and very high–resolution satellite imagery. Fig 1A shows a one-degree square of northern Laos. The colored pixels indicate clearings in different years between 2000 and 2014 as recorded in the GFC data set [8]. Fig 1B to Fig 1E show examples of different zoom levels used to decide whether the pattern in the GFC data is indeed related to shifting cultivation Fig 1E (showing pattern of clearing for the current year of cultivation and different stages of fallow) or not Fig 1D (larger scale clearings with young rubber). The imagery used for illustrative purpose in Fig 1 is based on Copernicus Sentinel 2 data from 2016. Maps created in QGIS 2.16.

To approximate the current extent of shifting cultivation landscapes globally we used the results of a time-series analysis of mainly Landsat images characterizing forest extent and change [8], hereafter referred to as Global Forest Change (GFC) data set. The spatio-temporal pattern of the annual deforestation data from 2000 to 2014 at a resolution of 30 meters provides the basis for our approximation. Given that biomass regrows very quickly in the humid tropics, the GFC data set treats large shares of a field cleared for shifting cultivation and kept fallow for a relatively short period as deforestation. In addition, we used available very high–resolution satellite imagery from Bing and Google (most images dating from the period between 2008 and 2015; visited between September and April 2015) in an ArcGIS Desktop 10.4 and QGIS environment to examine visually whether a given area for which the GFC data indicated a spatio-temporal pattern of small-scale clearings consistent with shifting cultivation, was indeed likely to be under shifting cultivation. This is only the case if, in addition to a pattern of small-scale clearings in the GFC data, a spatio-temporal pattern of different stages of fallow and regrowth is visible in the very high–resolution imagery from Bing and Google. Fig 1 illustrates the procedure we used for this visual inspection. By zooming into areas where these clearings indicate possible shifting cultivation, we were able to determine visually whether they were accompanied by the pattern of fallows characteristic of shifting cultivation (Fig 1E) or not (Fig 1D). As the data we used (GFC as well as Bing and Google imagery) cover the period from 2000 to 2014 and 2015, respectively, our assessment of the current extent of shifting cultivation does not relate to any specific year. We attribute it to 2010 for the sake of simplicity.

As our aim was to provide, in a timely manner, a global-scale overview of landscapes showing signs of shifting cultivation for use in global land use–related earth system modeling scenarios [27], we worked at an aggregated level using one-degree cells, which corresponds approximately to the scale of current earth system model analyses. Initially, we considered all 6,704 one-degree cells covering the land area between 30°S and 30°N, where shifting cultivation is likely to occur [17]. From this, we excluded regions where shifting cultivation can safely be assumed to not have been present for centuries (e.g. Australia, the Gulf States, arid areas in Africa) or where it disappeared several decades ago (e.g. Peninsular Malaysia, central and southern Thailand) [28]. This left us with 2,817 one-degree cells, which we then further investigated.

Using the data and approach described above, each one-degree cell was examined visually at various zoom levels (roughly 1:100’000 but, if necessary, occasionally at larger scales) to determine whether it showed the very specific spatio-temporal signature of shifting cultivation (see Fig 1). Visual interpretation has well-known limitations in terms of subjectivity and potentially limited reproducibility [29], but there are two main reasons why a visual approach has been chosen: Firstly, while a number of approaches has recently been developed to detect shifting cultivation based on automated approaches at the regional and national level using remote sensing data (e.g. [30–37]) such approaches cannot yet be up-scaled to global level due to data availability as well as computational limitations. Secondly, the detection and monitoring of complex shifting cultivation mosaics using automated remote sensing approaches remains challenging [33,38,39] and the mentioned small spatio-temporal signature of vegetation clearings and regrowth is very specific to shifting cultivation and visual interpretation is therefore suitable [40]. Moreover, even if GFC deforestation data processing using the Google Earth engine might enable this automation in the future, the visual approach will still be highly valuable for validating the robustness of automated approaches.

To get a first estimation of the occurrence of shifting cultivation, we classified each cell under investigation into one of five shifting-cultivation occurrence classes: none, very low, low, moderate, or high. We did not perform any detailed spatial delineation of the actual area under shifting cultivation, as the goal of our study was to provide a global one degree–gridded product. Hence, the occurrence level was estimated and not measured and the classification was based on a coarse assessment of the landscape (also see accuracy assessment below). The five classes corresponded to the following rough ranges of area shares of shifting cultivation landscapes (currently cultivated fields plus all stages of fallows) within an entire one-degree cell: none: < 1%; very low: 1–9%; low: 10–19%; moderate: 20–39%; high: ≥ 40%. The area approximation of actual shifting cultivation landscapes was performed based on the average occurrence rates in the one-degree cells for each of the five classes above (>1% class: 0%; 1–9%: 5%; 10–19%: 15%; 20–39%: 30%; ≥40%:70%). The area calculation was done within a Mollweide projection. We compared this result to Butler’s (1980) binary (presence or absence) map of shifting cultivation, gridded into one-degree cells.

**Fig 2:**
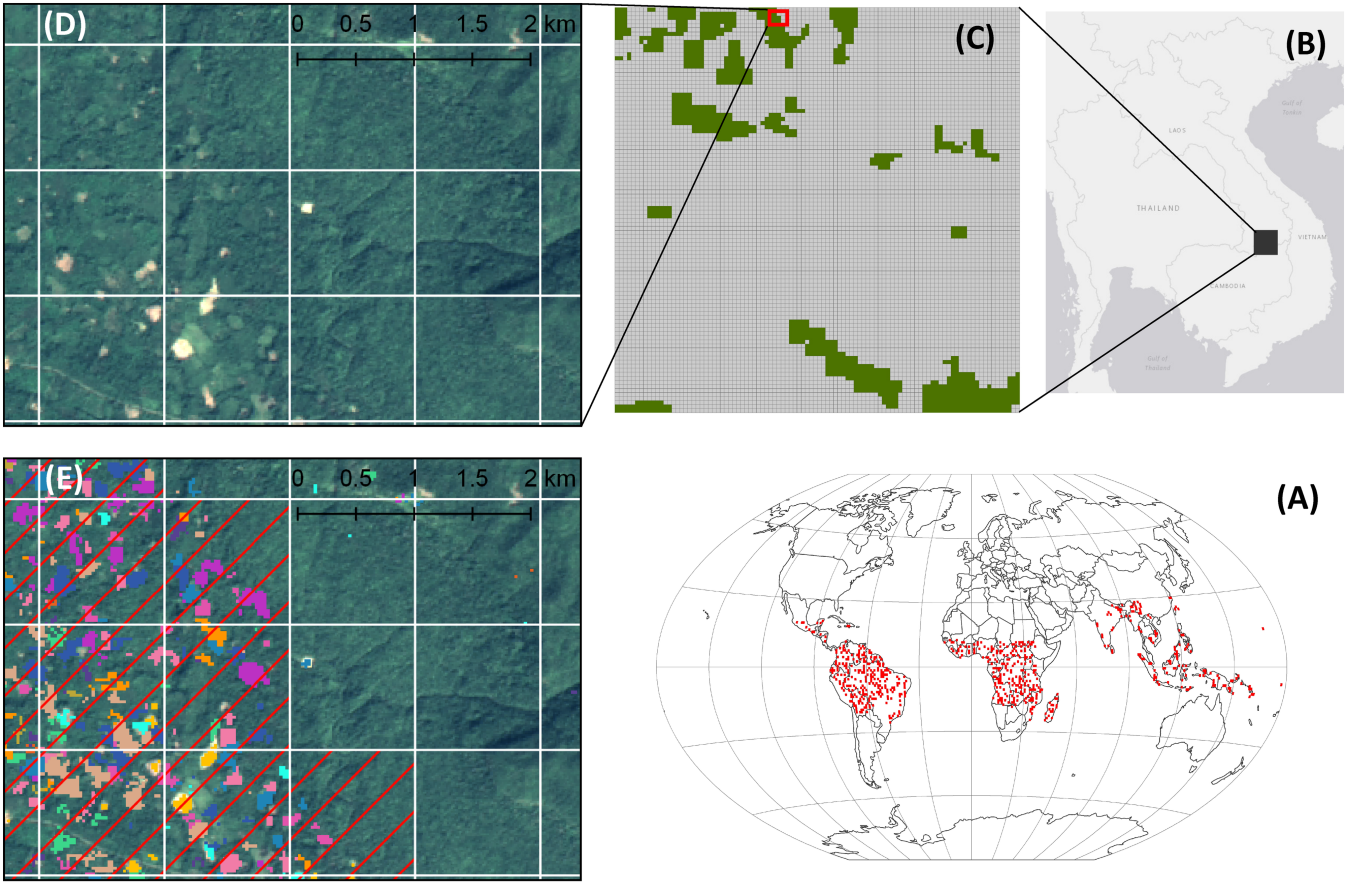
Creation of validation dataset. (Fig 2A): the global distribution of the stratified sample of the 328 one-degree cells used in the validation data set. (Fig 2B): Location of the one-degree cell of Fig 2C - 2E. (Fig 2C): One-degree cell with a mesh of 1/100 degree cells as a basic unit for the validation data set, green cells having a shifting cultivation occurrence class of >1% in our global classification. The red box marks the extent of Fig 2D and Fig 2E. (Fig 2DA) and (Fig 2E): The white line grid marks the 1/100 degree cells used as basic unit for the validation data. Based on the spatio-temporal pattern of the GFC data (different colours denoted different year of clearings) and the patterns of clearing and regrowth in the very high resolution imagery (here Bing), a 1/100-degree cell is being classified as showing shifting cultivation or not. The red hatching in (B) indicates the 1/100 degree cells that were classified as having shifting cultivation. (Source of imagery in 2D and 2E: Pansharpened Landsat 8 image, acquisition date January 5 2014, available from the U.S. Geological Survey.). Maps created in QGIS 2.16.

The validation of shifting cultivation mapping is generally challenging due to the lack of reference data [41,42]. Using recent regional and national automated classification of shifting cultivation as reference (e.g. [30–37]) would be insufficient, as they only cover very few of our one-degree cells and are not representative globally. In addition, the methods used in the different national and regional assessments vary greatly and are far from being standardized. As global level ground data collection is not feasible and no global data on shifting cultivation for the considered time span of 2000 to 2014 is available, we generated a validation dataset, which contains a detailed delineation of the areas under shifting cultivation for a stratified sample of one-degree cells. The stratified validation sample design was chosen considering the distribution of the validation samples per occurrence class and the spatial distribution per continents. Taking into consideration the stratification criteria, 328 one-degree cells were randomly selected to be validated. As for the entire global level assessment of all the 2,817 one degree cells considered, the GEFC and available very high resolution imagery (i.e. Bing and Google Earth) were used. In addition, to the extent multi-temporal very high resolution imagery was available on Google Earth, these were used to get an even better impression of the landscape and its land-use dynamics. According to Olofsson et al. [43] when using the same source material for the classification as for the reference or validation data, it is essential to create the reference/validation data with a more accurate process than the classification. Each of the 328 sample one-degree cells was subdivided in cells of 1/100 degree by 1/100 degree, resulting in 10,000 verification cells per one-degree cell. Each of these samples was visually examined in detail at scale of 1:20,000 or lower for the presence or absence of the above mentioned shifting cultivation specific spatio-temporal signs of clearing and regrowth on the landscape. To assess the accuracy of our estimated occurrence levels of shifting cultivation on the landscape, this validation data was used to calculate the actual area shares of the 1/100 by 1/100 one-degree cells classified as having shifting cultivation in validation data for all 328 one degree samples cells. Fig 2 illustrates this process for one sample cell. In this specific case 1088 or 10.88% of the 1/100 degree cells within the one-degree sample cell were detected as having shifting cultivation in the validation data sets. In our classification the occurrence for shifting cultivation was estimated at a “low level”, meaning 10–19%, which in this case was in line with the validation data set. The producer’s and user’s accuracies were calculated for each occurrence class (< 1%; very low: 1–9%; low: 10–19%; moderate: 20–39%; high: ≥ 40%.) and the overall accuracy and the Cohen’s kappa coefficient were calculated.

### 2.3 Past and future change in areas under shifting cultivation

In order to assess both past and future land use transitions in areas currently under shifting cultivation, we identified experts with recent knowledge of these areas by searching the Web of Science (“All Databases”) for papers published between 2005 and 2015 whose title contained “shifting cultivation” or similar terms. We used a search string similar to the one used for the literature review:

[Title]: "Shifting cultivation" or swidden or "slash-and-burn" or "slash and burn" or "shifting agriculture" AND [Year published]: 2005–2015

The search was performed in September 2015 and generated 316 results. After eliminating papers that were not related to recent shifting cultivation in the tropics (e.g. archeological studies or historical studies of shifting cultivation in Europe), duplicates, and papers whose authors had deceased in the meantime, 282 papers remained. The 270 first authors of these papers were listed and their email addresses were found.

**Fig 3:**
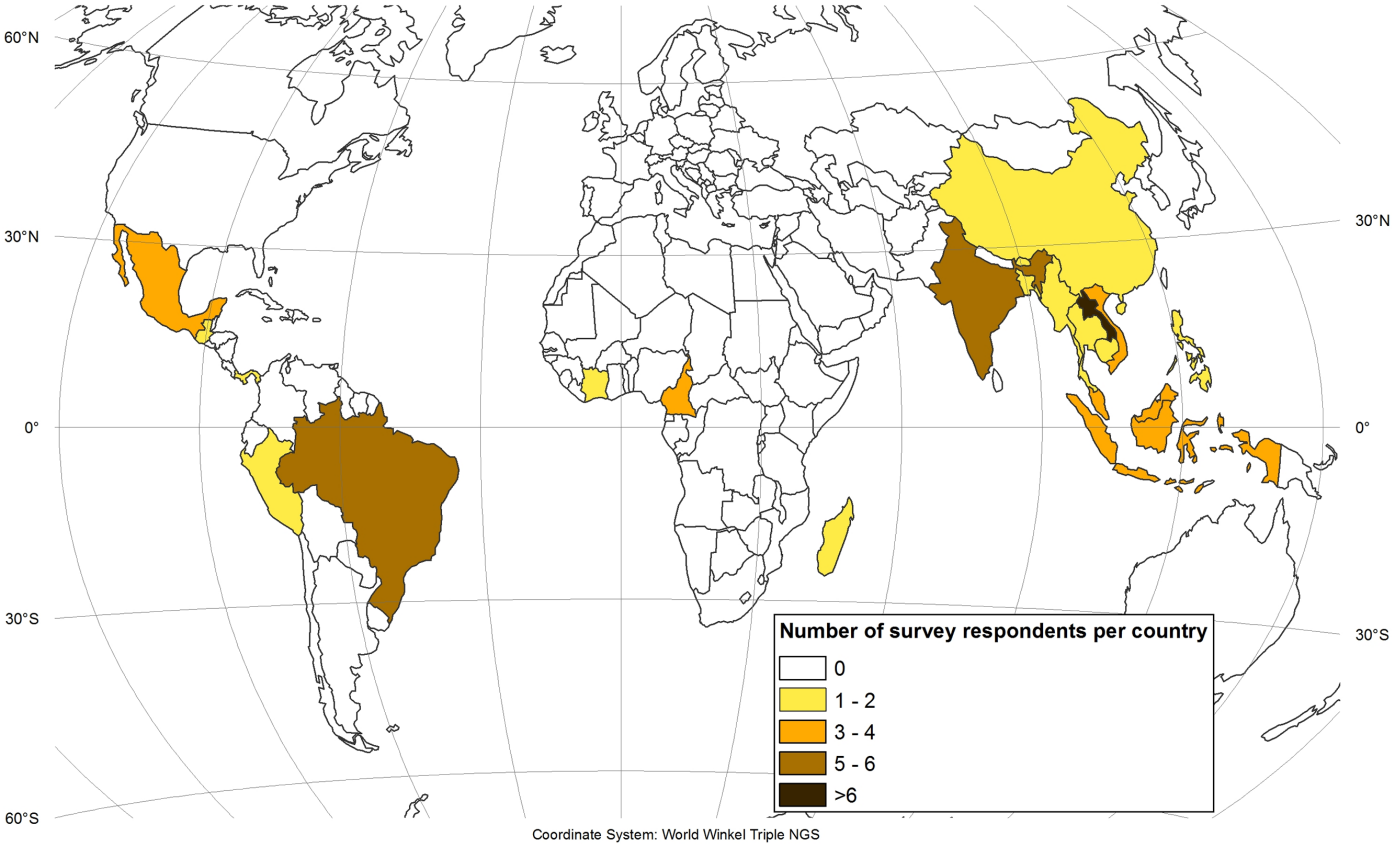
Survey responses received, by country of study. This figure was elaborated by the first author using ArcGIS 10.4.

An online questionnaire was designed and sent to these 270 authors in September 2015 using survey monkey. All answers were anonymous and cannot be traced back to the individual expert. Thirty-eight email addresses were no longer functional and 7 authors declined to participate for various reasons. Of the remaining 225 authors, 72 responded and 49 provided usable information (see S1 File). There was a bias towards responses from researchers who worked in Southeast Asia (see Fig 3); but this is also the world region where most research on shifting cultivation has been done, whereas Africa has the fewest studies and is clearly under-represented in light of the considerable occurrence of shifting cultivation there [17,44].

The respondents were asked to estimate the current spatial extent of shifting cultivation as well as past and future trends in the development of this extent in their area of interest. The most important questions included:

- Indicate shifting cultivation area changes in the following periods of the past (no change, expansion, decline, disappearance): 1900–1970; 1970–2000; 2000–2015.
- Indicate how you expect the shifting cultivation occurrences to change in the future for the following periods (no change, expansion, decline, disappearance): 2015–2030; 2030–2060; 2060–2090.

The information provided by the respondents related to very different spatial scales, ranging from village to district, provincial, and, in some cases, national scales. Moreover, it is not possible to know in detail how the respondents arrived at their assessment of past and future extents of shifting cultivation. The selection process ensured that all are experts in the field, but there may be disciplinary or personal differences in the way that especially the future of shifting cultivation was assessed.

With these caveats in mind, we aggregated responses to the national scale and to three supranational regions: the tropical parts of 1) Central and South America, 2) Africa, and 3) Asia. We then made an estimate of trends in occurrence of shifting cultivation for these aggregated regions for 2030, 2060 and 2090 also taking into account the historical trends between the Butler map and our 2010 classification. It is important to note that these are indeed very rough possible scenarios and should be seen as expected trends rather than fixed percentages of decline.

To generate a spatially explicit prediction of the temporal dynamics (decline) of shifting cultivation through to the 2090s, we combined the survey results with several simple assumptions. We initiated occurrence at a one-degree resolution in 2010 (base year) at the mean of the above-stated ranges for each occurrence category in the assessment of current landscape with signs of shifting cultivation based on the GFC data (c.f. 2.2). For each grid cell, the occurrence of shifting cultivation declined linearly by the mid-point of the estimated losses in 2030, 2060 and 2090 (see Section 3.4). If and when the occurrence of shifting cultivation in a grid cell fell below 5% (mean value for “very low”), shifting cultivation was assumed to disappear in that grid cell. If the survey provided information about when (i.e. 2030, 2060, 2090) shifting cultivation was expected to disappear completely from a specific country (n = 21), all one-degree cells having their centroid within that country where classified as having zero occurrence of shifting cultivation after that time, regardless of the above-described gradual decrease.

## 3. Results and discussion

### 3.1 Early spatial representations of shifting cultivation

**Fig 4:**
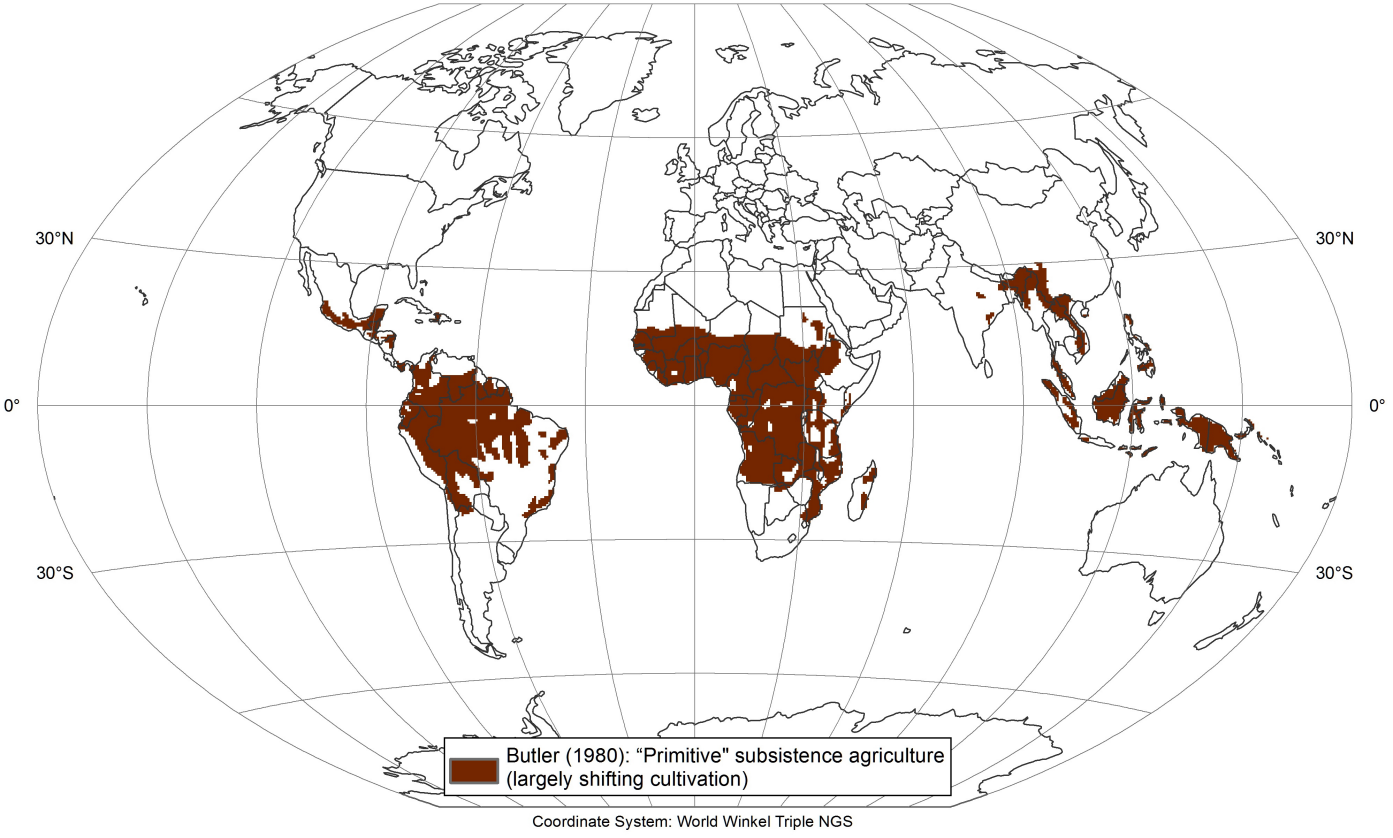
Butler (1980) map of areas where land use includes “primitive subsistence agriculture,” which in the humid tropics largely consists of shifting cultivation (reproduction by first author using ArcGIS 10.4 based on Hurtt et al. [2]).

The aforementioned map produced by Butler [13] (Fig 4) is a hand-drawn representation of areas where both shifting and non-shifting “primitive subsistence agriculture exists,” as Butler put it. The map shows large areas under such agriculture in Africa, tropical Central and South America, and Southeast Asia. No source is reported for the map, but most likely it builds on a number of regional studies that was carried out between the 1940s and the 1970s, as well as “general knowledge” of where shifting cultivation and other extensive smallholder farming systems were found. The map is binary (presence-absence), with no information on occurrence frequency or land-use intensity.

At the regional scale, an assessment by Spencer [45] provides a slightly more differentiated picture of the presence of shifting cultivation practices in Asia. The data sources are not entirely clear, but the assessment seems to be based on a number of studies carried out between the end of the 19th century and the late 1950s, along with expert judgement. Spencer’s hand-drawn map is largely in agreement with Butler’s for most areas, but it shows larger areas under shifting cultivation in Thailand, Myanmar, India, and Sri Lanka. Spencer [45] indicates an approximate area under shifting cultivation (currently cultivated fields plus all stages of fallows) of around 110 million hectares (Mha) for Asia. The classic studies on shifting cultivation in Africa [46–48] do not provide any area data for the continent, and for Central and South America we were unable to find any regional-scale past area estimation.

In the absence of further data to validate past estimations of the extent of shifting cultivation, the Butler map may be considered a reasonable representation of the global distribution of shifting cultivation from 1960 into the 1970s. Although the areas shown on the map also include non-shifting forms of subsistence agriculture, it is reasonable to assume that shifting cultivation predominated in the humid and sub-humid tropics, which are the main focus of the present study.

### 3.2 Recent and current extents of shifting cultivation

Our review of the more recent literature revealed surprisingly few studies containing regional or global estimates of areas under shifting cultivation. A study conducted by the Food and Agriculture Organization (FAO) of the United Nations in 1985 (based on 90 tropical countries reporting forest fallow areas within the FAO/UNEP Tropical Forest Resources Assessment Project 1982 [49]) estimated the worldwide extent of shifting cultivation in the early 1980s at 400 Mha [50], and an assessment made in 2011 with the aim of estimating greenhouse gas emissions arrived at 260 Mha for the 2000s [1]. An area of 1,000 Mha was mentioned by Davidson et al. [51] citing Sanchez et al. [24], who in turn had cited Dixon et al. [52]; the latter source, however, does not provide this information, so the 1,000 Mha claim seems to be unfounded.

The study by Silva et al. [1] used the Global Land Cover 2000 (GLC2000) data set [53] and the Map of the Ecosystems for Central America [54] to estimate the extent of shifting cultivation at the global scale. This approach, however, suffers from the shortcoming that land cover data are of very limited use in estimating land use practices, which is acknowledged by Silva et al. [1]. While shifting cultivation’s signature on the landscape may be captured as a mixture or mosaic of agriculture and forest land cover classes, this alone does not suffice to indicate with certainty the presence of shifting cultivation. For example, the large areas of tree plantations established in Vietnam and southern China in the 1980s and 1990s can also leave a mosaic signature at a certain point in time and might therefore be incorrectly interpreted as shifting cultivation (Fig 1). On the other hand, large areas with shifting cultivation in Central Africa, for example in the Democratic Republic of the Congo [36], are strongly under-represented in Silva et al. [1], possibly owing to the scale (1-km resolution) of the GLC2000 data sets.

The number of estimates of areas under shifting cultivation at regional and national scales in the literature is also very limited. For Southeast Asia, Schmidt-Vogt et al. [10] compiled available published shifting cultivation area estimates for seven countries: Thailand, Laos, Cambodia, Vietnam, Myanmar, Malaysia, and Indonesia. However, the areas indicated for each country vary greatly depending on the source. For example, data for Laos indicate between 2 and over 6 Mha, while more recent figures based on remote sensing (multi-temporal Landsat) for northern Laos—where the largest share of shifting cultivation in the country is found—are 3.1 Mha [33] and 2.6 Mha [34]. Comparison of these figures with the GLC2000-based area estimate for Laos of almost 11 Mha [1] underlines the problem of using the GLC2000 to estimate areas under shifting cultivation. For India, Goswami et al. [55], citing the Wasteland Atlas [56], estimated the extent of shifting cultivation in the mid-2000s at 5.6 Mha (only area under cultivation), whereas the GLC2000-based estimate is 7.6 Mha [1]. Regarding Central and South America, the only available source [57] used the secondary forest class as defined in FAO’s Forest Resource Assessment 1990 [58] as a proxy and stated the area under shifting cultivation to be 165 Mha. Silva et al. [1] indicate 110 Mha for this region, but unlike FAO they did not include Mexico in their estimation. As for Africa, we found only one recent national study, on the Democratic Republic of the Congo [36]. Its authors detected changes in what they call “the rural complex” for the period from 2000 to 2010. The areas referred to as “the rural complex” may be used as a proxy for the presence of shifting cultivation. The authors estimated that these areas made up 13.1% of the country’s total land area in 2010; assuming the Democratic Republic of the Congo has a land area of 2.27 million km2 [59], this would amount to nearly 30 Mha, compared to 16 Mha based on the global GLC2000 data set in Silva et al. [1].

**Fig 5:**
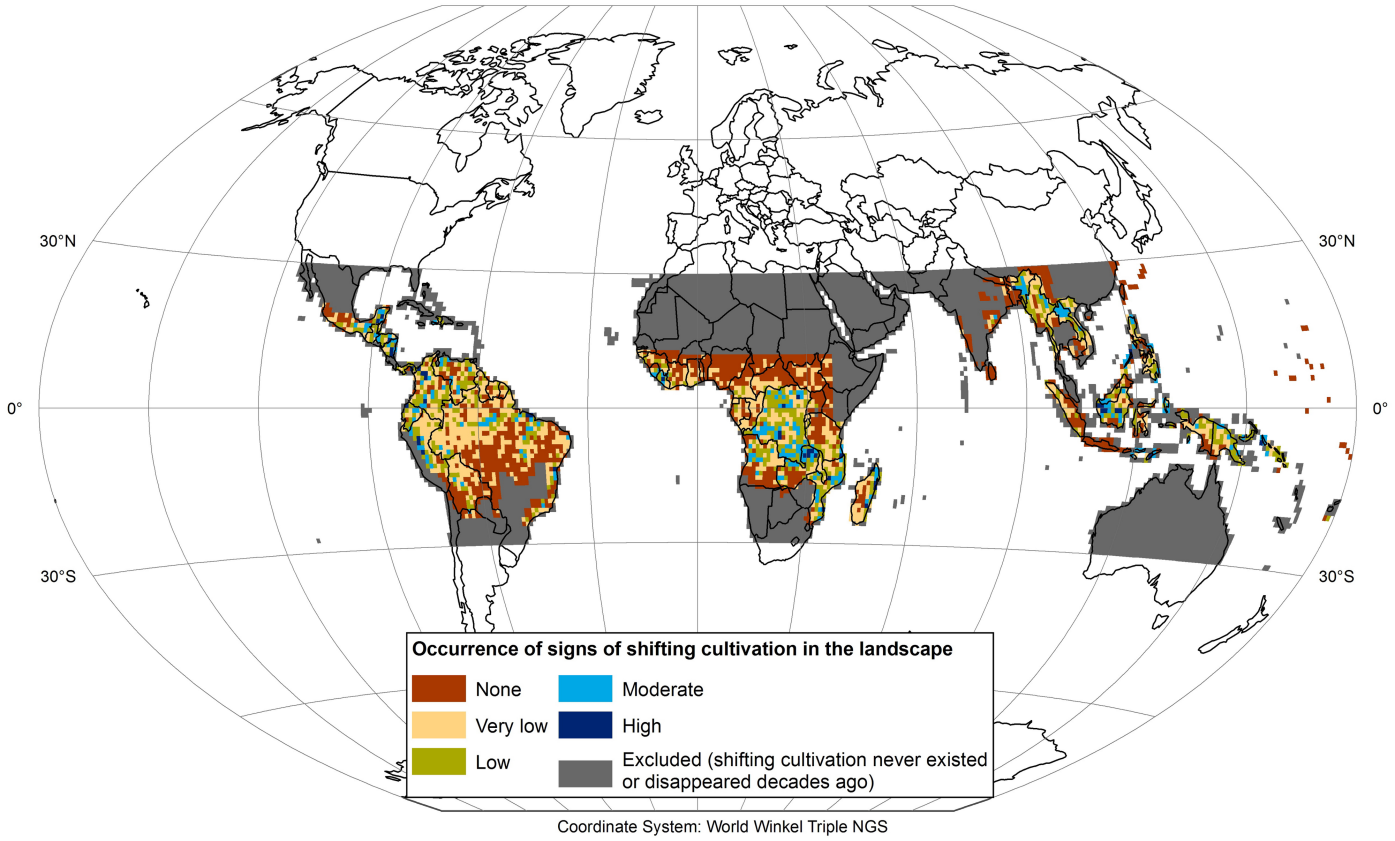
Estimation of landscapes showing signs of shifting cultivation around 2010 between 30°S to 30°N. Based on visual inspection of annual global deforestation data [8] from 2000 to 2014 and very high-resolution satellite imagery. Areas in which shifting cultivation can be assumed to have never existed or disappeared decades ago have been excluded from the analysis (dark gray). This figure was elaborated by the first author using ArcGIS 10.4.

Fig 5 presents the results of our own visual approximation of the global extent of shifting cultivation around 2010 at a one-degree resolution, based on Hansen et al.’s (2013) GFC data and very high–resolution satellite imagery. The map shows that shifting cultivation is still present across large areas of the humid tropics. However, the occurrence of shifting cultivation within most of the individual one-degree cells is very low, meaning that it is a minor component of the overall landscape. More widespread signs of shifting cultivation were found mostly in small pockets, with the exception of larger areas in Central Africa (e.g. northern Zambia and in the Democratic Republic of the Congo), parts of southeastern Africa (e.g. Mozambique), northern mainland Southeast Asia (northern Laos and Myanmar), Borneo, and, to a lesser degree, Central America, Colombia, and Peru.

Globally, sixty-two per cent of the investigated one-degree cells showed signs of shifting cultivation, with surprisingly similar shares across the 3 regions, ranging between 59 and 65% (Table 1) In absolute terms, the majority of cells with shifting cultivation are located in the Americas and Africa (almost 78%).

**Table 1:** Numbers and percentages of one-degree cells studied that showed signs of shifting cultivation (SC) or not (No SC), as well as percentages of cells showing signs of shifting cultivation in the various occurrence classes, per region. The area of interest ranges from 30°S and 30°N (6,704 one-degree cells on landmass), while the area investigated includes 2,817 cells. The remaining cells (3,887) were excluded from the analysis as shifting cultivation can be assumed to have never existed or disappeared decades ago (see Fig 5 and Method section).

Region	Total (cells)	No SC # cells (%)	SC # cells (%)	Very low SC (% of SC)	Low SC (% of SC)	Moderate SC (% of SC)	High SC (% of SC)
Central and South America	1,082	375 (35%)	707 (65%)	59.7	27.9	9.9	2.5
Africa	1,082	432 (40%)	650 (60%)	52.0	30.8	14.6	2.6
Asia	653	267 (41%)	386 (59%)	43.0	37.0	17.1	2.8
**Total**	**2,817**	**1074 (38%)**	**1743 (62%)**	**53.1**	**31.0**	**13.3**	**2.6**

The very low occurrence of shifting cultivation within a majority of cells, particularly in the Americas, points towards shifting cultivation being either a form of cultivation practiced in landscapes where only a minor share of the land is used for agriculture (e.g. in the Amazon and parts of the Democratic Republic of the Congo), or a residual form of cultivation in landscapes that have mostly been transformed to other land uses (such as permanent agriculture or tree crops, e.g. in parts of Southeast Asia).

The validation of the estimation of landscapes showing signs of shifting cultivation revealed that 95.1% of the one-degree cells showing signs of shifting cultivation in the validation data correspond to the results in our classification. Also when considering the different levels of occurrence of shifting cultivation estimated based on the overall impression of the landscape per one-degree cell, the accuracies were high (see confusion matrix in Table 2), with an overall classification accuracy of 87.8%. This indicates that, despite the subjectivity involved on estimating the landscape level of occurrence of shifting cultivation in our classification, the method led to reproducible and accurate results. The comparably low user and producer accuracies of the class “moderate” occurrence (20–39% coverage of shifting cultivation landscapes of the entire one-degree cell) is not surprising as it could have been expected that this intermediate class would be the most difficult one to estimate.

**Table 2:** Accuracy assessment of the global classification (Fig 5).

	**Validation data**							**PA**
**Classification**	**Classes**	**None**	**Very Low**	**Low**	**Moderate**	**High**	**Total**	
	**None**	153	7	0	0	0	160	95.6%
	**Very Low**	9	68	2	1	0	80	85.0%
	**Low**	0	9	43	7	0	59	72.8%
	**Moderate**	0	0	1	16	3	20	80.0%
	**High**	0	0	0	1	8	9	88.8%
	**Total**	162	84	46	25	11	328	
**UA**		94.4%	80.9%	93.5%	64.0%	72.7%		

The overall accuracy is 87.8% and Cohen’s Kappa is 0.816 (PA = producer’s accuracy; UA = user’s accuracy).

While an area approximation of actual shifting cultivation landscapes based on our analysis is difficult due to the estimated and not measured shared of shifting cultivation of each one-degree cell we believe that, given the high accuracy of this estimation (see Table 2), a conservative estimate can nonetheless be derived by visually inspecting the cells and allocating reasonable shares of shifting cultivation landscape (currently cultivated fields plus all stages of fallows) to them as described in the methods section. Using the mean values of the ranges specified for the different occurrence classes for all cells in all classes results in a total area of 280 Mha. This number certainly requires further validation before it can be claimed to be an adequate estimate of the global area under shifting cultivation. Nonetheless, it is more evidence-based than the 1,000 Mha of unclear origin that are repeatedly cited in the literature (e.g. [24]). Surprisingly, our estimate is not too far from the 259 Mha proposed in Silva et al [1], even though their estimate excludes large areas under shifting cultivation and includes areas under other forms of agriculture and natural vegetation. At the regional scale, the only number to which we can compare our result is the 110 Mha for Asia estimated by Spencer [45], which is considerably larger than our estimate for this region of approximately 70 Mha. The difference appears plausible if we consider that Spencer’s estimate is based on a hand-drawn map and that Asia has seen considerable decreases in shifting cultivation since that map was created (see section on recent trends below).

### 3.3 Recent trends in the extent of shifting cultivation

Two steps enabled us to gain insights into larger recent (last 40 to 50 years) trends in the development of the global area under shifting cultivation. First, we compared the Butler [13] map (Fig 4) with our own spatial estimate (Fig 5) of areas that were under shifting cultivation around 2010. Second, we combined the results of this comparison with those of our expert survey.

**Fig 6:**
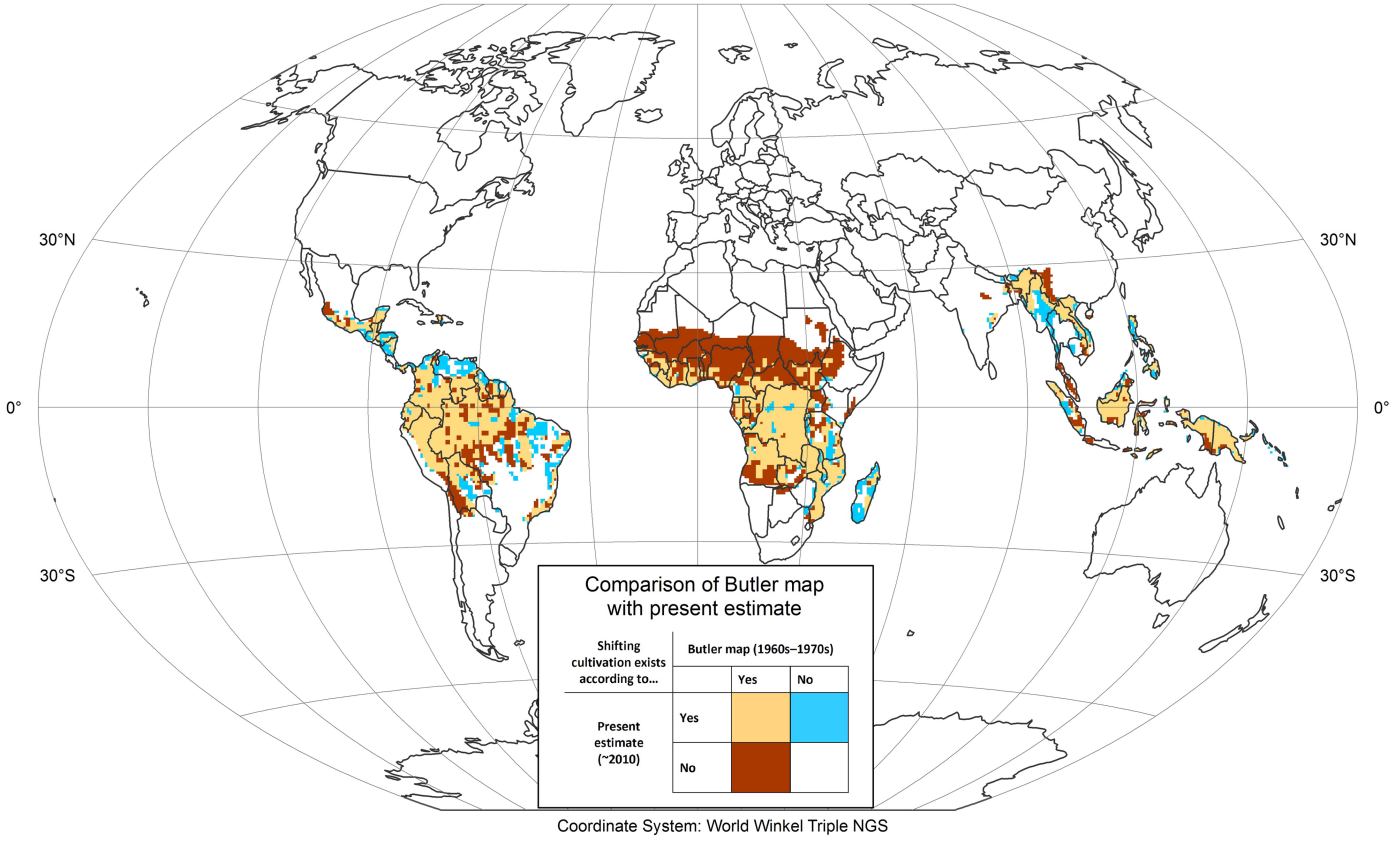
Comparison of the Butler map [13] (showing the status in the 1960s to 1970s) with our results (showing the status around 2010). This figure was elaborated by the first author using ArcGIS 10.4.

The difference between the Butler map—which is presumably based on studies dating from the 1960s and 1970s [13]—and our own current investigation around 2010 is displayed in Fig 6. Generally, the two approximations of areas under shifting cultivation are in fairly good agreement, especially when considering that the large differences in the arid areas of northern Africa, southern Angola, and Zambia and the high-mountain areas of eastern Bolivia are largely explained by the fact that Butler included other, non-shifting forms of extensive smallholder agriculture and that shifting cultivation in these areas based on the climatic condition never could have been widespread. Many of the other decreases from Butler’s to our map (dark brown in Fig 6) can also most likely be related to the actual disappearance of shifting cultivation in these areas that have seen significant land use transitions over the past 40 to 50 years. This is the case for Peninsular Malaysia [28], parts of Sumatra [60], Yunnan Province in southern China [61]and the southern part of the Brazilian Amazon [62], for example. When looking at this comparison, it is important to keep in mind that only full “disappearance” of shifting cultivation within a one-degree cell will show as a change between the two data sets; gradual decreases—which normally precede full disappearance—are not captured if the occurrence of shifting cultivation in the respective cells according to our present estimate remains greater than 5%. For this reason, the known widespread decrease in the occurrence of shifting cultivation in Southeast Asia (e.g. Laos, Vietnam) over the past 15 to 25 years is not reflected in this comparison. The areas newly classified as having shifting cultivation (blue in Fig 6) are more likely to have been missed in the Butler map than to represent actual new areas under shifting cultivation, as many of these regions are well known to still have significant shifting cultivation landscapes. Such areas are found in parts of Southeast Asia (e.g. Myanmar [63–65]), Central America (e.g. Yucatan Peninsula [66], northern South America (e.g. Venezuela [67]), and Madagascar [68]. Consequently, if further research (e.g. within global land-use models) needs spatially explicit estimates of areas under shifting cultivation at intervals lying between the status shown in the Butler map (1960s to 1970s) and our estimations for 2010 (e.g. decadal), the areas newly classified as having shifting cultivation (blue in Fig 6) should be added to the original Butler map, thereby producing an updated estimated extent of shifting cultivation for this earlier period.

After analyzing the spatially differentiated changes between the status in the 1960s to 1970s as shown in the Butler map and our data for 2010 (Fig 6), we combined them with data from our expert survey about changes in areas under shifting cultivation between 1970 to 2000 and 2000 to 2010 as well as with information from the literature. On this basis, we can provide a preliminary overview of regional and national trends in the development of the extent of shifting cultivation over the past 40 to 50 years.

Regarding South and Southeast Asia, a meta-analysis by van Vliet et al. [17] showed that these regions have experienced marked decreases in shifting cultivation. These changes are not fully reflected in the above comparison between the Butler map and our own spatial investigation for 2010 because in many areas they have resulted only in a reduced occurrence of shifting cultivation but not yet in its full disappearance. However, the expert survey confirms that shifting cultivation has fully disappeared between the 1970s and the 2000s in various areas of mainland Southeast Asia, such as southern Thailand, Peninsular Malaysia, and China. In a second cluster of countries—Laos, Cambodia and Myanmar—shifting cultivation areas have decreased drastically since 2000. The “increase” of shifting cultivation in Myanmar shown in Fig 6 is due to a marked underestimation in the Butler map, which can possibly be explained by the limited availability of information about Myanmar after the military government came to power in 1962. For large parts of insular Southeast Asia and South Asia (e.g. India, Bangladesh), comparison of the maps (Fig 6) and the survey responses point to similar trends.

Trends in humid tropical Africa vary widely. In West and Central Africa, shifting cultivation is largely continuing and has even been expanding in certain areas. This trend, which was also identified by van Vliet et al. [17], contrasts with developments in other parts of Africa, where decreases and the disappearance of areas under shifting cultivation have dominated over the last two decades. This is reflected both in the comparison of the two spatial data sets (Fig 6) and in the survey responses. However, it is important to underline that this assessment is based on very few survey responses (Fig 3) due to the small number of existing studies on shifting cultivation in Africa. Furthermore, it should be noted that the large difference between the two spatial data sets (Fig 6) in arid and semiarid parts of Africa (Sahelian and Sudanian zones of northern Africa as well as parts of southern Africa) is partly due to the fact that Butler included other, non-shifting smallholder farming systems in his map. In large parts of humid West Africa, shifting cultivation is still widespread—with the exception of Nigeria, where it has all but disappeared and remains present only in small pockets. In Central Africa, shifting cultivation also remains very widespread, with an even higher occurrence than in West Africa; in certain areas it is still expanding, such as in the Democratic Republic of the Congo [36]. In eastern and southern Africa, shifting cultivation is still present, but not very common, with particularly low occurrences in Kenya and Tanzania. Madagascar has seen only slight decreases; especially along its eastern escarpment the area under shifting cultivation has remained stable over the last two decades (e.g. [68]).

In Central America, shifting cultivation is still widespread, and both Fig 6 and the survey results indicate an increase in some areas (e.g. Panama, Guatemala) well into the 2000s. In Mexico, however, the trend has been towards decreasing areas under shifting cultivation. South America shows mixed trends: Areas under shifting cultivation have clearly decreased in the southern Brazilian Amazon, whereas survey responses indicate that they are expanding in other parts of the Brazilian Amazon and in Peru. Despite the limited number of survey responses on Central and South America, it appears that here, unlike in Southeast Asia, areas under shifting cultivation have not seen a strong decline over the last 20 years.

### 3.4 Preliminary estimations of future trends in the extent of shifting cultivation

Predicting future trends in the development of any form of land use requires extreme caution [23,69]. However, future climate projections and carbon budget estimates on carbon land sinks and land use emissions based on earth system models depend on such predictions as input, so any attempt to provide an evidence base for them is certainly a worthwhile improvement compared to relying on historical trends or static futures. For this reason, we have estimated future changes in shifting cultivation by combining observed trends between the Butler map and our own map with expert’s survey responses regarding future changes in shifting cultivation in different parts of the world. Based on this we expect that shifting cultivation is likely to decrease significantly in all regions over the next 20 years, and we estimate that it will tend towards disappearance in all regions by 2090 (Table 3). In Asia, we expect that continued rapid economic development and the related changes in agricultural practices and, more importantly, in the economic structure (from the primary to the secondary and tertiary sectors) may cause shifting cultivation to disappear faster than in Africa or the Americas. By contrast, we expect that shifting cultivation will persist for a longer time in Africa, especially in Central Africa. Shifting cultivation tends to persist when population density is low and when options for agricultural development or alternative livelihoods are limited [22,70,71]. Both conditions apply to considerable parts of Central Africa.

**Table 3:** Estimated trends in area under shifting cultivation between 2010 and the 2030s, 2060s, and 2090s expressed in percentage ranges of losses compared to 2010. The ranges are based on the expert survey and observed trends between the Butler map and our 2010 classification (Fig 5).

Region	2030	2060	2090
Central and South America	10–30%	60–80%	80–100%
Africa	0–20%	60–80%	80–100%
Asia	30–50%	80–100%	100%

While keeping in mind the inherent limitations of these predictions, we can identify a number of more specific patterns. For large parts of Southeast Asia, the survey results point towards that the current swift decrease in shifting cultivation continues, and that a large share of the area under shifting cultivation will have disappeared by 2030, and the remaining pockets are likely to be almost entirely gone by 2060.Moreover, the survey results indicate trends for some specific Asian countries:

- Vietnam and Laos: Shifting cultivation is likely to be greatly reduced by 2030 and completely gone by 2060.
- Myanmar: Shifting cultivation is estimated to mostly disappear sometime between 2060 and 2090 if conflicts between union government and ethnic armed groups are resolved.
- Borneo and Sulawesi: Shifting cultivation is expected to disappear sometime between 2030 and 2060.
- India and Bangladesh: Shifting cultivation is estimated to disappear by 2030.
- Papua New Guinea: Shifting cultivation may persist well into the second half of this century, perhaps even until 2090.

Humid tropical Africa is probably the region for which developments are most difficult to predict due to limited data. The results indicate that shifting cultivation is likely to persist longest in Africa. In some specific areas, especially in Central Africa, it is likely to increase over the next decade before it begins to decline.

- West Africa (Sierra Leone, Liberia, Guinea, Côte d’Ivoire, Ghana): Shifting cultivation is anticipated to diminish rapidly by 2030 and to largely disappear by 2060 if peace is upheld and there is no major return of Ebola; but if conflicts resurface, shifting cultivation may persist well into the second half of this century.
- Central Africa: Shifting cultivation is estimated to persist well into the 2060s or longer due to the vast reserve of remote forested areas.
- Madagascar: Shifting cultivation is expected to remain widespread, especially along the eastern escarpment, until well beyond 2030.

For Central and South America, the survey shows a mixed picture, with some areas being quite stable or even likely to experience expansion in the near future, and other areas (e.g. Mexico and Brazil) likely to see a fairly rapid decrease and disappearance. Overall, a decrease after 2030 and disappearance after 2060 is expected.

**Fig 7:**
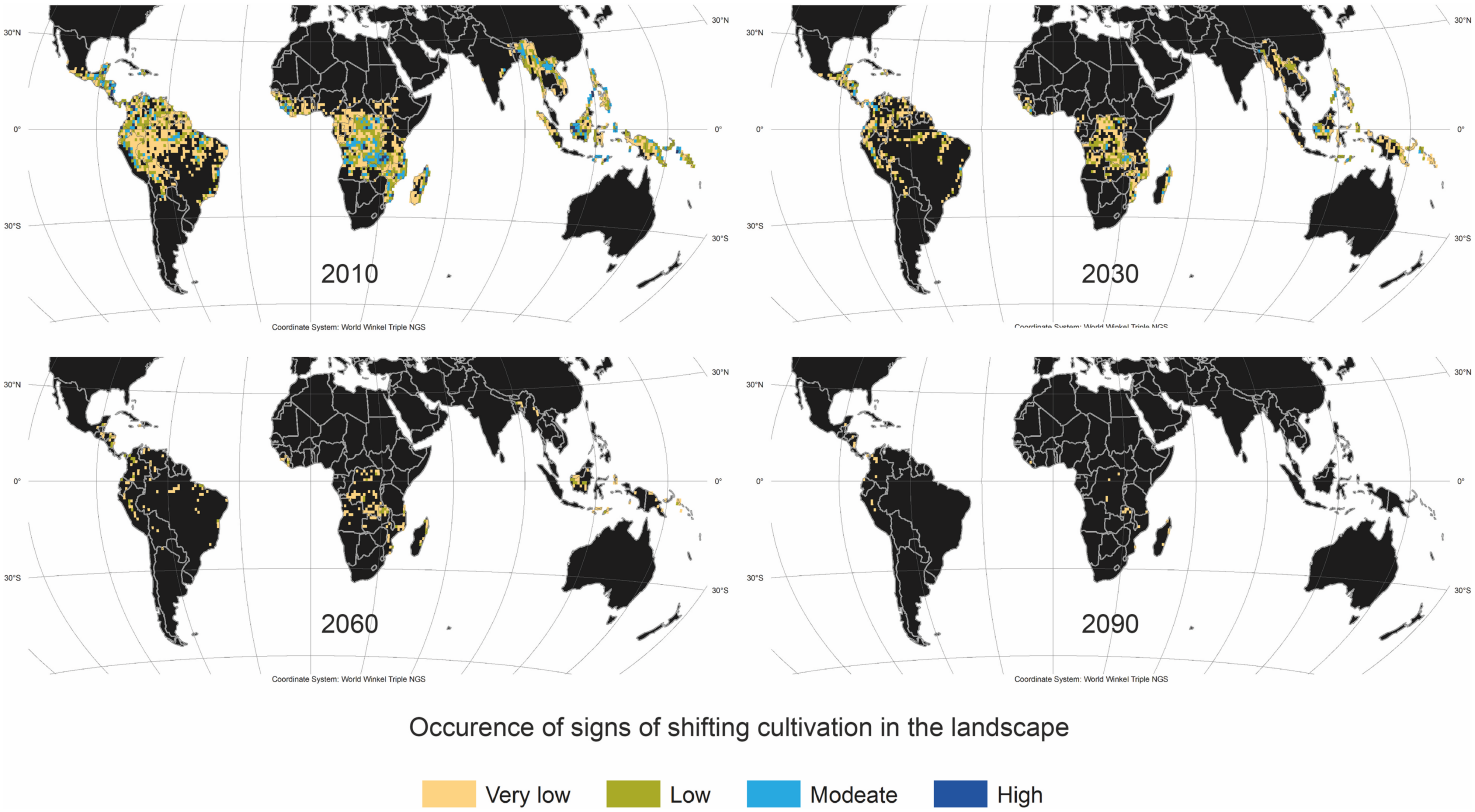
Preliminary estimates of changes in the occurrence of shifting cultivation between today and 2030, 2060 and 2090. This visualization is based on the estimation of landscapes showing signs of shifting cultivation around 2010 (Fig 5) as base year and estimated decreases of shifting cultivation (Table 3) based on the expert surveys and observed trend between the Butler map and our 2010. This figure was elaborated by the first author using ArcGIS 10.4.

Applying these largely survey-based estimates (Table 3) to our map of the extent of shifting cultivation in 2010 (Fig 5), we were able to visually display our rough predictions of developments in the extents of shifting cultivation by 2030, 2060, and 2090 (Fig 7). We would like to emphasize that the maps in Fig 7 are indeed very coarse estimates of the future global or continental extent of shifting cultivation—and not an exact representation of where precisely it might be found in the near or far future. We have nonetheless ventured to display our estimates in predictive maps because they are based on a spatially explicit analysis in 2010. We believe that this represents a significant improvement on the shifting cultivation predictions that have been used so far in global land use models to estimate future greenhouse gas emissions, and we hope that our estimates can be a valuable input for future comparisons between models and international synthesis studies such as the upcoming Coupled Model Intercomparison Project Phase 6 (CMIP6) experiments [27] and the next Assessment Report of the Intergovernmental Panel on Climate Change (IPCC).

## 4. Conclusions

Given the unavailability of automated approaches to detect shifting cultivation at a global level and deliver data in a timely manner for ongoing earth system modeling, we have used a visual interpretation approach to detect shifting cultivation. Based on existing data and knowledge, we have made a first attempt at estimating possible future trends in the distribution of shifting cultivation until 2090. While our estimates are based on non-automated methods and expert information from different parts of the world, we argue that our work nonetheless advances the state of knowledge considerably, especially with regard to earth system modeling scenarios, which have proved sensitive to the inclusion of shifting cultivation and up to now have used shifting cultivation data based on a hand drawn map from the 1980’s.

Shifting cultivation remains widespread, despite decreases in its extent over the last four to five decades. However, we found that its occurrence in most one-degree cells, where it existed, was fairly limited, with roughly 85% of these cells showing occurrence levels below 20% (currently cultivated fields and all stages of fallows). Our cautious estimation indicates that the global extent of shifting cultivation, including currently cultivated fields and all stages of fallows, may amount to roughly 280 Mha, with the largest share in Africa, followed by the Americas and Asia.

Our preliminary estimated for the future indicate that the area under shifting cultivation is expected to shrink considerably over the next decades. This raises issues of livelihood security and resilience among people currently depend on shifting cultivation, who may face reduced provision of ecosystem services and limited access to land due to the expansion of permanent agriculture, tree plantations, urban areas, and forest protection or restoration [18,19,44,72]. However, this future may also provide better opportunities for production and income generation if development efforts are sensitive to the needs of shifting cultivators [73,74]. According to our tentative predictions, shifting cultivation—which has been a globally important form of human crop cultivation for millennia—may be gone by the end of this century.

Improvements in mapping the extent of shifting cultivation and trends in its development may be expected in the near future. Researchers are currently developing automated approaches that are capable of processing decades of Landsat data and detecting the spatio-temporal patterns of shifting cultivation. The recently launched Sentinel-2 instruments with their augmented repeat frequency will generally help to improve remote sensing–based analyses of the humid tropics, which are complicated by frequent and persistent cloud cover. Analysis of Landsat data back to Landsat4 (launched in 1982) could provide more than 30 years of pan-tropical records, covering a time of significant change in the distribution of shifting cultivation across the humid tropics and perhaps also in the length of fallow periods. These approaches, however, are still in the making and will require substantial resources. The present study is a first step towards a future in which we will know more about the global distribution of shifting cultivation; we hope it opens the door to quantifying shifting cultivation’s importance for local as well as the global socio-ecological systems.

## Supporting information

S1 File Complied results of expert survey responses. (PDF)

## References

[1] Silva JMN, Carreiras JMB, Rosa I, Pereira JMC. Greenhouse gas emissions from shifting cultivation in the tropics, including uncertainty and sensitivity analysis. J Geophys Res. 2011;116.

[2] Hurtt GC, Chini LP, Frolking S, Betts RA, Feddema J, Fischer G, et al Harmonization of land-use scenarios for the period 1500–2100: 600 years of global gridded annual land-use transitions, wood harvest, and resulting secondary lands. Clim Change. 2011;109: 117–161. doi: 10.1007/s10584-011-0153-2

[3] Houghton RA, House JI, Pongratz J, van der Werf GR, DeFries RS, Hansen MC, et al Carbon emissions from land use and land-cover change. Biogeosciences. 2012;9: 5125–5142.

[4] Brovkin V, Boysen L, Arora VK, Boisier JP, Cadule P, Chini L, et al Effect of Anthropogenic Land-Use and Land-Cover Changes on Climate and Land Carbon Storage in CMIP5 Projections for the Twenty-First Century. J Clim. 2013;26: 6859–6881.

[5] Ellis EC, Kaplan JO, Fuller DQ, Vavrus S, Klein Goldewijk K, Verburg PH. Used planet: A global history. Proc Natl Acad Sci. 2013;110: 7978–7985. doi: 10.1073/pnas.1217241110 23630271 10.1073/pnas.1217241110 PMC3657770

[6] Monfreda C, Ramankutty N, Foley JA. Farming the planet: 2. Geographic distribution of crop areas, yields, physiological types, and net primary production in the year 2000. Glob Biogeochem Cycles. 2008;22: n/a–n/a.

[7] Klein Goldewijk K, Beusen A, van Drecht G, de Vos M. The HYDE 3.1 spatially explicit database of human-induced global land-use change over the past 12,000 years. Glob Ecol Biogeogr. 2011;20: 73–86.

[8] Hansen MC, Potapov PV, Moore R, Hancher M, Turubanova SA, Tyukavina A, et al High-Resolution Global Maps of 21st-Century Forest Cover Change. Science. 2013;342: 850–853. doi: 10.1126/science.1244693 24233722 10.1126/science.1244693

[9] Padoch C, Coffey K, Mertz O, Leisz SJ, Fox J, Wadley RL. The demise of swidden in Southeast Asia? Local realities and regional ambiguities. Geogr Tidsskr-Dan J Geogr. 2007;107: 29–41.

[10] Schmidt-Vogt D, Leisz SJ, Mertz O, Heinimann A, Thiha T, Messerli P, et al An Assessment of Trends in the Extent of Swidden in Southeast Asia. Hum Ecol. 2009;37: 269–280. doi: 10.1007/s10745-009-9239-0

[11] Frolking S, Palace MW, Clark DB, Chambers JQ, Shugart HH, Hurtt GC. Forest disturbance and recovery: A general review in the context of spaceborne remote sensing of impacts on aboveground biomass and canopy structure. J Geophys Res. 2009;114 doi: 10.1029/2008JG000911

[12] Mertz O, Birch-Thomsen T, Elberling B, Rothausen S, Bruun TB, Reenberg A, et al Changes in shifting cultivation systems on small Pacific islands. Geogr J. 2012;178: 175–187. doi: 10.1111/j.1475-4959.2011.00447.x

[13] Butler JH. Economic Geography: Spatial and Environmental Aspects of Economic Activity. New York: John Wiley; 1980.

[14] Mertz O, Padoch C, Fox J, Cramb RA, Leisz SJ, Lam NT, et al Swidden Change in Southeast Asia: Understanding Causes and Consequences. Hum Ecol. 2009;37: 259–264. doi: 10.1007/s10745-009-9245-2

[15] Mertz O, Müller D, Sikor T, Hett C, Heinimann A, Castella J-C, et al The forgotten D: challenges of addressing forest degradation in complex mosaic landscapes under REDD+. Geogr Tidskr—Dan J Geogr. 2012;112: 63–76.

[16] Rudel TK, Schneider L, Uriarte M, Turner BL, DeFries R, Lawrence D, et al Agricultural intensification and changes in cultivated areas, 1970–2005. Proc Natl Acad Sci U S A. 2009;106: 20675–20680. doi: 10.1073/pnas.0812540106 19955435 10.1073/pnas.0812540106 PMC2791618

[17] van Vliet N, Mertz O, Heinimann A, Langanke T, Pascual U, Schmook B, et al Trends, drivers and impacts of changes in swidden cultivation in tropical forest-agriculture frontiers: A global assessment. Glob Environ Change-Hum Policy Dimens. 2012;22: 418–429. doi: 10.1016/j.gloenvcha.2011.10.009

[18] Cramb RA, Colfer CJP, Dressler W, Laungaramsri P, Le QT, Mulyoutami E, et al Swidden Transformations and Rural Livelihoods in Southeast Asia. Hum Ecol. 2009;37: 323–346. doi: 10.1007/s10745-009-9241-6

[19] Fox J, Fujita Y, Ngidang D, Peluso N, Potter L, Sakuntaladewi N, et al Policies, Political-Economy, and Swidden in Southeast Asia. Hum Ecol. 2009;37: 305–322. doi: 10.1007/s10745-009-9240-7 19609457 10.1007/s10745-009-9240-7 PMC2709851

[20] Thongmanivong S, Fujita Y, Phanvilay K, Vongvisouk T. Agrarian Land Use Transformation in Northern Laos: From Swidden to Rubber. Southeast Asian Stud. 2009;47. Available: kyoto-seas.org/pdf/47/3/470306.pdf

[21] Vongvisouk T, Mertz O, Thongmanivong S, Heinimann A, Phanvilay K. Shifting cultivation stability and change: Contrasting pathways of land use and livelihood change in Laos. Appl Geogr. 2014;46: 1–10. doi: 10.1016/j.apgeog.2013.10.006

[22] Mertz O, Leisz SJ, Heinimann A, Rerkasem K, Thiha, Dressler W, et al Who Counts? Demography of Swidden Cultivators in Southeast Asia. Hum Ecol. 2009;37: 281–289. doi: 10.1007/s10745-009-9249-y

[23] Müller D, Sun Z, Vongvisouk T, Pflugmacher D, Xu J, Mertz O. Regime shifts limit the predictability of land-system change. Glob Environ Change. 2014;28: 75–83. doi: 10.1016/j.gloenvcha.2014.06.003

[24] Sanchez PA, Palm CA, Vosti SA, Tomich T, Kasyoki J. Alternatives to slash and burn. Challenges and approaches of an international consortium In: Palm CA, Vosti SA, Sanchez PA, Ericksen PJ, editors. Slash-and-Burn Agriculture The Search for Alternatives. Columbia University Press New York; 2005 pp. 3–37.

[25] Crains M. Voices from the Forest: Integrating Indigenous Knowledge into Sustainable Upland Farming. Washington D.C.: RFF Press; 2007.

[26] Crains M. Shifting Cultivation and Environmental Change: Indigenous People, Agriculture and Forest Conservation. London: Earthscan; 2015.

[27] Eyring V, Bony S, Meehl GA, Senior C, Stevens B, Stouffer RJ, et al Overview of the Coupled Model Intercomparison Project Phase 6 (CMIP6) experimental design and organisation. Geosci Model Dev Discuss. 2015;8: 10539–10583. doi: 10.5194/gmdd-8-10539-2015

[28] Rasul G, Thapa B. Shifting cultivation in the mountains of South and Southeast Asia: regional patterns and factors influencing the change. Land Degrad Dev. 2003;14: 495–508.

[29] Lu D, Mausel P, Brondizio E, Moran E. Change detection techniques. Int J Remote Sens. 2004;25: 2365–2407.

[30] Castella J-C, Lestrelin G, Hett C, Bourgoin J, Fitriana YR, Heinimann A, et al Effects of Landscape Segregation on Livelihood Vulnerability: Moving From Extensive Shifting Cultivation to Rotational Agriculture and Natural Forests in Northern Laos. Hum Ecol. 2013;41: 63–76.

[31] Hett C, Castella J-C, Heinimann A, Messerli P, Pfund J-L. A landscape mosaics approach for characterizing swidden systems from a REDD+ perspective. Appl Geogr. 2012;32: 608–618. doi: 10.1016/j.apgeog.2011.07.011

[32] Hurni K, Hett C, Epprecht M, Messerli P, Heinimann A. A Texture-based land cover classification for the delineation of a shifting cultivation landscape in the Lao PDR using landscape metrics. Remote Sens. 2013;5: 3377–3396.

[33] Hurni K, Hett C, Heinimann A, Messerli P, Wiesmann U. Dynamics of Shifting Cultivation Landscapes in Northern Lao PDR Between 2000 and 2009 Based on an Analysis of MODIS Time Series and Landsat Images. Hum Ecol. 2013;41: 21–36. doi: 10.1007/s10745-012-9551-y

[34] Liao C, Feng Z, Li P, Zhang J. Monitoring the spatio-temporal dynamics of swidden agriculture and fallow vegetation recovery using Landsat imagery in northern Laos. J Geogr Sci. 2015;25: 1218–1234. doi: 10.1007/s11442-015-1229-0

[35] Messerli P, Heinimann A, Epprecht M. Finding Homogeneity in Heterogeneity—A New Approach to Quantifying Landscape Mosaics Developed for the Lao PDR. Hum Ecol. 2009;37: 291–304. doi: 10.1007/s10745-009-9238-1 19609458 10.1007/s10745-009-9238-1 PMC2709899

[36] Molinario G, Hansen M, Potapov P. Forest cover dynamics of shifting cultivation in the Democratic Republic of Congo: a remote sensing-based assessment for 2000–2010. Environ Res Lett. 2015;10: 94009.

[37] Zähringer JG, Hett C, Ramamonjisoa B, Messerli P. Beyond deforestation monitoring in conservation hotspots: Analysing landscape mosaic dynamics in north-eastern Madagascar. Appl Geogr. 2016;68: 9–19.

[38] DeFries R, Achard F, Brown S, Herold M, Murdiyarso D, Schlamadinger B, et al Earth observations for estimating greenhouse gas emissions from deforestation in developing countries. Environ Sci Policy. 2007;10: 385–394. doi: 10.1016/j.envsci.2007.01.010

[39] Li P, Feng Z, Jiang L, Liao C, Zhang J. A Review of Swidden Agriculture in Southeast Asia. Remote Sens. 2014;6: 1654–1683. doi: 10.3390/rs6021654

[40] Leisz SJ, N thi Thu Ha, N thi Bich Yen, Lam NT, Vien TD. Developing a methodology for identifying, mapping and potentially monitoring the distribution of general farming system types in Vietnam’s northern mountain region. Agric Syst. 2005;85: 340–363. doi: 10.1016/j.agsy.2005.06.015

[41] Langner A, Miettinen J, Siegert F. Land cover change 2002–2005 in Borneo and the role of fire derived from MODIS imagery. Glob Change Biol. 2007;13: 2329–2340. doi: 10.1111/j.1365-2486.2007.01442.x

[42] Li P, Feng Z. Extent and Area of Swidden in Montane Mainland Southeast Asia: Estimation by Multi-Step Thresholds with Landsat-8 OLI Data. Remote Sens. 2016;8: 44 doi: 10.3390/rs8010044

[43] Olofsson P, Foody GM, Herold M, Stehman SV, Woodcock CE, Wulder MA. Good practices for estimating area and assessing accuracy of land change. Remote Sens Environ. 2014;148: 42–57. doi: 10.1016/j.rse.2014.02.015

[44] Mukul SA, Herbohn J. The impacts of shifting cultivation on secondary forests dynamics in tropics: A synthesis of the key findings and spatio temporal distribution of research. Environ Sci Policy. 2016;55: 167–177. doi: 10.1016/j.envsci.2015.10.005

[45] Spencer J.E: Shifting Cultivation in Southeastern Asia. Berkeley: Berkeley: University of California Press; 1966.

[46] de Schlippe P. Shifting Cultivation in Africa: the Zande System of Agriculture. London: Routledge and Kegan Paul; 1956.

[47] Nye PH, Greenland DJ. The Soil Under Shifting Cultivation. Harpenden, UK: Commonwealth Bureau of Soils, Agricultural Bureau; 1960.

[48] Miracle MP. Agriculture in the Congo Basin Tradition and Change in African Rural Economies. Madison, Milwaukee: The University of Wisconsin Press; 1967.

[49] FAO/UNDP. Tropical forest resources. FAO; 1982.

[50] Lanly JP. Defining and measuring shifting cultivation. Unasylva. 1985;37: 17–21.

[51] Davidson EA, de Abreu Sa TD, Reis Carvalho CJ, de Oliveria Figueiredo R, Kato M do SA, Kato OR, et al An integrated greenhouse gas assessment of an alternative to slash-and-burn agriculture in eastern Amazonia. Glob Change Biol. 2008;14: 998–1007. doi: 10.1111/j.1365-2486.2008.01542.x

[52] Dixon J, Gulliver A, Gibbon D. Farming Systems and Poverty—Improving Farmers’ Livelihoods in a Changing World [Internet]. Rome: Food & Agriculture Organization of the UN (FAO); 2001 ftp://ftp.fao.org/docrep/fao/003/y1860e/y1860e00.pdf

[53] Bartholomé E, Belward AS. GLC2000: a new approach to global land cover mapping from Earth observation data. Int J Remote Sens. 2005;26: 1959–1977. doi: 10.1080/01431160412331291297

[54] Vreugdenhil, Meerman A, Meyrat L, Gomez D, Graham D J. Map of the Ecosystems of Central America: Final Report. Washington D.C: World Bank; 2002.

[55] Goswami K, Choudhury HK, Saikia J. Factors influencing farmers’ adoption of slash and burn agriculture in North East India. For Policy Econ. 2012;15: 146–151.

[56] Wastelands Atlas. Categorywise and districtwise wasteland area. Ministry of Rural Development. National Remote Sensing Agency. Government of India; 2010.

[57] de Jong W, Freitas L, Baluarte J, van de Kop P, Salazar A, Inga E, et al Secondary forest dynamics in the Amazon floodplain in Peru. For Ecol Manag. 2001;150: 135–146.

[58] FAO. Forest Resource Assessment 1990. Rome: FAO; 1996.

[59] Central Intelligence Agency. In: The World Factbook. Washington, DC: Central Intelligence Agency [Internet]. 2016 [cited 9 Feb 2016]. https://www.cia.gov/library/publications/the-world-factbook/geos/cg.html

[60] Lawrence D, Peart DR, Leighton M. The impact of shifting cultivation on a rainforest landscape in West Kalimantan: spatial and temporal dynamics. Landsc Ecol. 1998;13: 135–148.

[61] Jianchu X, Fox J, Vogler JB, Yongshou ZPF, Lixin Y, Jie Q, et al Land-Use and Land-Cover Change and Farmer Vulnerability in Xishuangbanna Prefecture in Southwestern China. Environ Manage. 2005;36: 404–413. doi: 10.1007/s00267-003-0289-6 15995894 10.1007/s00267-003-0289-6

[62] Galford GL, Melillo J, Mustard JF, Cerri CEP, Cerri CC. The Amazon Frontier of Land-Use Change: Croplands and Consequences for Greenhouse Gas Emissions. Earth Interact. 2010;14: 1–24. doi: 10.1175/2010EI327.1

[63] Thein HM, Minn Y. Recovery process of fallow vegetation in the tradi-tiona l Karen swidden cultivation system in the Bago mountain range, Myanmar. Southeast Asian Stud. 2007;45: 317–333.

[64] Leimgruber P, Kelly DS, Steininger MK, Brunner J, MüLler T, Songer M. Forest cover change patterns in Myanmar (Burma) 1990–2000. Environ Conserv. 2005;32: 356 doi: 10.1017/S0376892905002493

[65] Htwe TN, Brinkmann K, Buerkert A. Spatio-temporal assessment of soil erosion risk in different agricultural zones of the Inle Lake region, southern Shan State, Myanmar. Environ Monit Assess. 2015;187. 26350794 10.1007/s10661-015-4819-5

[66] Schmook B. Shifting maize cultivation and secondary vegetation in the Southern Yucatán: successional forest impacts of temporal intensification. Reg Environ Change. 2010;10: 233–246. doi: 10.1007/s10113-010-0128-2

[67] Yerena E, Padrón J, Vera R, Martínez Z, Bigio D. Building Consensus on Biological Corridors in the Venezuelan Andes. Mt Res Dev. 2003;23: 215–218. doi: 10.1659/0276-4741(2003)023[0215:BCOBCI]2.0.CO;2

[68] Zaehringer J, Eckert S, Messerli P. Revealing Regional Deforestation Dynamics in North-Eastern Madagascar—Insights from Multi-Temporal Land Cover Change Analysis. Land. 2015;4: 454–474. doi: 10.3390/land4020454

[69] Rounsevell MDA, Pedroli B, Erb K-H, Gramberger M, Busck AG, Haberl H, et al Challenges for land system science. Land Use Policy. 2012;29: 899–910. doi: 10.1016/j.landusepol.2012.01.007

[70] Mertz O. The relationship between length of fallow and crop yields in shifting cultivation: a rethinking. Agrofor Syst. 2002;55: 149–159.

[71] Heinimann A, Hett C, Hurni K, Messerli P, Epprecht M, Jørgensen L, et al Socio-Economic Perspectives on Shifting Cultivation Landscapes in Northern Laos. Hum Ecol. 2013;41: 51–62. doi: 10.1007/s10745-013-9564-1

[72] Bruun TB, de Neergaard A, Lawrence D, Ziegler AD. Environmental Consequences of the Demise in Swidden Cultivation in Southeast Asia: Carbon Storage and Soil Quality. Hum Ecol. 2009;37: 375–388. doi: 10.1007/s10745-009-9257-y

[73] van Vliet N, Adams C, Guimaraes Vieira IC, Mertz O. “Slash and Burn” and “Shifting” Cultivation Systems in Forest Agriculture Frontiers from the Brazilian Amazon. Soc Nat Resour. 2013;26: 1454–1467. doi: 10.1080/08941920.2013.820813

[74] Mertz O, Bruun T. Shifting cultivation policies in Southeast Asia. A need to work with, rather than against, smallholder farmers In: Crains M, editor. Shifting Cultivation Policies: Balancing Environmental and Social Sustainability. CABI; in press.

