# Supplementary material for: A global view of shifting cultivation: Recent, current, and future extent
Source: PLoS One. 2017 Sep 8;12(9):e0184479. doi: 10.1371/journal.pone.0184479 (PMC5590965; doi:10.1371/journal.pone.0184479)
Supplement: S1 File — (PDF) [file pone.0184479.s001.pdf]

S1 file : Summary of expert survey results

Table 1. Overall summary

Number of respondents	72
Number of continents	5
Number of countries	23

Table 2. Regions & Countries

Regions (Continents)	Number of countries
Southeast Asia (incl. China)	9
South Asia	3
North America	1
South America	2
Central America	3
Africa	4
Europe	1

Table 3. Southeast Asia – including China

Country (No. of respondents)	How has the area under S.C. changed 2000-2015?	How has the area under S.C. changed 1970-2000?	How has the area under S.C. changed 1900-1970?	How has the area under S.C. changed 2000-2030?	How has the area under S.C. changed 2030-2060?	How has the area under S.C. changed 2060-2090?
**Laos (11)**	Decline, smaller areas are now under S.C.	Expansion, S.C. areas stable ->or increased during that period	No change, stable -> expansion	Decline, areas under S.C. will have decreased compared to today	Decline, areas under S.C. will have decreased or disappeared compared to 2030	Disappearance Or declined
**Vietnam (3)**	Expansion, larger areas are now under S.C.	Expansion, S.C. on areas increased during that period	No change, stable -> expansion	Decline, areas under S.C. will have decreased compared to today	Decline, areas under S.C. will have decreased compared to 2030 -> disappearance	Disappearance -> decline

Country (No. of respondents)	How has the area under S.C. changed 2000-2015?	How has the area under S.C. changed 1970-2000?	How has the area under S.C. changed 1900-1970?	How has the area under S.C. changed 2000-2030?	How has the area under S.C. changed 2030-2060?	How has the area under S.C. changed 2060-2090?
	Expansion, larger areas are now under S.C.	Expansion, S.C. areas increased during that period	Expansion, S.C. areas increased during that period	Decline, areas under S.C. will have decreased compared to today	No change expected compared to 2030	No change expected compared to 2060
**Cambodia (2)**	Decline, smaller areas are now under shifting cultivation	No change, stable	No change, stable	Decline, areas under S.C. will have decreased compared to today	Decline, areas under S.C. will have decreased compared to 2030	Decline, areas under S.C. will have decreased compared to 2060
**Thailand (1)**	No valid data	No valid data	No valid data	No valid data	No valid data	No valid data
**Indonesia (3)**	Decline, smaller areas are now under S.C.	From expansion to decline in other areas of Kalimantan	No change, stable	From decline in some areas to Disappearance in other areas	No change expected compared to 2030	No change expected compared to 2060
**Malaysia (3)**	Decline, smaller areas are now under S.C.	Decline, S.C. areas decreased during that period	Expansion, S.C. areas increased during that period	Decline, areas under S.C. will have decreased compared to today	Disappearance	Disappearance – around 2040!
**Philippines (1)**	Decline, smaller areas are now under S.C.	Decline, S.C. areas decreased during that period	Expansion, S.C. areas increased during that period	Decline, areas under S.C. will have decreased compared to today	Decline, areas under S.C. will have decreased compared to 2030	Decline, areas under S.C. will have decreased compared to 2060
**China (2)**	Disappearance	Decline, S.C. areas decreased during that period	Expansion, S.C. areas increased during that period	Disappearance	Disappearance	Disappearance
**Myanmar (1)**	Decline, smaller areas are now under shifting cultivation	Decline, shifting cultivation areas decreased during that period	Expansion, shifting cultivation areas increased during that period	Decline, areas under shifting cultivation will have decreased compared to today	Decline, areas under shifting cultivation will have decreased compared to 2030	Disappearance

Table 4. South Asia

Country (No. of respondents)	How has the area under S.C. changed 2000-2015?	How has the area under S.C. changed 1970-2000?	How has the area under S.C. changed 1900-1970?	How has the area under S.C. changed 2000-2030?	How has the area under S.C. changed 2030-2060?	How has the area under S.C. changed 2060-2090?
**India (6)**	Ranging from no changes, stable – to decline – to disappearance	Expansion, shifting cultivation areas increased during that period	Expansion, shifting cultivation areas increased during that period	Decline, areas under S.C. will have decreased compared to today	Decline – to disappearance - areas under S.C. will have decreased compared to 2030	Decline – to disappearance - areas under S.C. will have decreased compared to 2030
**Bangladesh (1)**	-	-	-	-	-	-
**Bhutan (1)**	Decline, smaller areas are now under shifting cultivation	Decline, shifting cultivation areas decreased during that period	No change, stable	Disappearance	No change expected compared to 2030	No change expected compared to 2060

Table 5. South America

Country (No. of respondents)	How has the area under S.C. changed 2000-2015?	How has the area under S.C. changed 1970-2000?	How has the area under S.C. changed 1900-1970?	How has the area under S.C. changed 2000-2030?	How has the area under S.C. changed 2030-2060?	How has the area under S.C. changed 2060-2090?
**Brazil (5)**	No changes, stable	Expansion, shifting cultivation areas increased during that period	I'm unable to assess whether there has been changes during that period	Expansion, areas under shifting cultivation will be larger compared to today	No change expected compared to 2030	Decline, areas under shifting cultivation will have decreased compared to 2060
	No changes, stable	Decline, shifting cultivation areas decreased during that period	Expansion, shifting cultivation areas increased during that period	Decline, areas under shifting cultivation will have decreased compared to today	Decline, areas under shifting cultivation will have decreased compared to 2030	Decline, areas under shifting cultivation will have decreased compared to 2060
	Decline, smaller areas are now	No change, stable	Expansion, shifting cultivation areas	Decline, areas under shifting cultivation	Expansion, areas under shifting	Expansion, areas under shifting cultivation will

Country (No. of respondents)	How has the area under S.C. changed 2000-2015?	How has the area under S.C. changed 1970-2000?	How has the area under S.C. changed 1900-1970?	How has the area under S.C. changed 2000-2030?	How has the area under S.C. changed 2030-2060?	How has the area under S.C. changed 2060-2090?
	under shifting cultivation		increased during that period	will have decreased compared to today	cultivation will be larger compared to 2030	be larger compared to 2060
	Decline, smaller areas are now under shifting cultivation	No change, stable	Expansion, shifting cultivation areas increased during that period	Decline, areas under shifting cultivation will have decreased compared to today	Decline, areas under shifting cultivation will have decreased compared to 2030	No change expected compared to 2060 (if disappeared before 2060
	No changes, stable	Decline, shifting cultivation areas decreased during that period	Expansion, shifting cultivation areas increased during that period	Decline, areas under shifting cultivation will have decreased compared to today	Decline, areas under shifting cultivation will have decreased compared to 2030	Disappearance
**Peru (2)**	Expansion, larger areas are now under shifting cultivation	Expansion, shifting cultivation areas increased during that period	Expansion, shifting cultivation areas increased during that period	Expansion, areas under shifting cultivation will be larger compared to today	Expansion, areas under shifting cultivation will be larger compared to 2030	Expansion, areas under shifting cultivation will be larger compared to 2060

Table 6. Central America

Country (No. of respondents)	How has the area under S.C. changed 2000-2015?	How has the area under S.C. changed 1970-2000?	How has the area under S.C. changed 1900-1970?	How has the area under S.C. changed 2000-2030?	How has the area under S.C. changed 2030-2060?	How has the area under S.C. changed 2060-2090?
**Panama (1)**	Expansion, larger areas are now under shifting cultivation	Expansion, shifting cultivation areas increased during that period	Expansion, shifting cultivation areas increased during that period	Expansion, areas under shifting cultivation will be larger compared to today		
**Guatemala (1)**	Expansion, larger areas are now under shifting cultivation	Expansion, shifting cultivation areas increased during that period	Expansion, shifting cultivation areas increased during that period	No change expected compared to today, stable		

Country (No. of respondents)	How has the area under S.C. changed 2000-2015?	How has the area under S.C. changed 1970-2000?	How has the area under S.C. changed 1900-1970?	How has the area under S.C. changed 2000-2030?	How has the area under S.C. changed 2030-2060?	How has the area under S.C. changed 2060-2090?
**Mexico (3)**	Decline, smaller areas are now under shifting cultivation	No change, stable	I'm unable to assess whether there has been changes during that period	Decline, areas under shifting cultivation will have decreased compared to today	Disappearance	Disappearance
	No changes, stable	Expansion, shifting cultivation areas increased during that period	-	-	-	-
	Decline, smaller areas are now under shifting cultivation	No change, stable	I'm unable to assess whether there has been changes during that period	Decline, areas under shifting cultivation will have decreased compared to today	Disappearance	Disappearance

Table 7. Africa

Country (No. of respondents)	How has the area under S.C. changed 2000-2015?	How has the area under S.C. changed 1970-2000?	How has the area under S.C. changed 1900-1970?	How has the area under S.C. changed 2000-2030?	How has the area under S.C. changed 2030-2060?	How has the area under S.C. changed 2060-2090?
**Cameroon (3)**	I'm unable to assess whether there has been changes or not	No change, stable	I'm unable to assess whether there has been changes during that period	Expansion, areas under shifting cultivation will be larger compared to today	Expansion, areas under shifting cultivation will be larger compared to 2030	Expansion, areas under shifting cultivation will be larger compared to 2060
	Expansion, larger areas are now under shifting cultivation	Expansion, shifting cultivation areas increased during that period	Expansion, shifting cultivation areas increased during that period	Expansion, areas under shifting cultivation will be larger compared to today	No change expected compared to 2030	No change expected compared to 2060
	Decline, smaller areas are now under shifting cultivation	Expansion, shifting cultivation areas increased during that period	Expansion, shifting cultivation areas increased during that period	Decline, areas under shifting cultivation will have decreased compared to today	Decline, areas under shifting cultivation will have decreased compared to 2030	Decline, areas under shifting cultivation will have decreased compared to 2060

Country (No. of respondents)	How has the area under S.C. changed 2000-2015?	How has the area under S.C. changed 1970-2000?	How has the area under S.C. changed 1900-1970?	How has the area under S.C. changed 2000-2030?	How has the area under S.C. changed 2030-2060?	How has the area under S.C. changed 2060-2090?
**Ivory Coast (1)**	Expansion, larger areas are now under shifting cultivation	Expansion, larger areas are now under shifting cultivation	Expansion, shifting cultivation areas increased during that period	Expansion, shifting cultivation areas increased during that period	Expansion, areas under shifting cultivation will be larger compared to 2030	Expansion, areas under shifting cultivation will be larger compared to 2060
**Madagacar (1)**	Expansion, larger areas are now under shifting cultivation	I'm unable to assess whether there has been changes or not during this period	I'm unable to assess whether there has been changes during that period	Expansion, areas under shifting cultivation will be larger compared to today	Expansion, areas under shifting cultivation will be larger compared to 2030	
